# The Effect of Growth Hormone on the Clinical Outcomes of Poor Ovarian Reserve Patients Undergoing *in vitro* Fertilization/Intracytoplasmic Sperm Injection Treatment: A Retrospective Study Based on POSEIDON Criteria

**DOI:** 10.3389/fendo.2019.00775

**Published:** 2019-11-12

**Authors:** Mei-hong Cai, Lin-zhi Gao, Xiao-yan Liang, Cong Fang, Yao-qiu Wu, Xing Yang

**Affiliations:** ^1^Reproductive Medical Center, The Sixth Affiliated Hospital of Sun Yat-Sen University, Guangzhou, China; ^2^Department of Reproductive Medical Center, Guangzhou First People's Hospital, School of Medicine, South China University of Technology, Guangzhou, China

**Keywords:** growth hormone, poor ovarian reserve, poor ovarian responders, clinical outcome, POSEIDON criteria

## Abstract

The aim of this retrospective analysis is to explore whether growth hormone (GH) pretreatment is beneficial for patients with poor ovarian reserve undertaking *in vitro* fertilization/intracytoplasmic sperm injection (IVF/ICSI) treatment. Poor ovarian reserve patients with anti-Mullerian hormone (AMH) <1.2 ng/mL were recruited and divided into the GH adjuvant group (GH+ group) and the counterpart without GH pretreatment (GH- group). One-to-one case-control matching was performed to adjust essential confounding factors between the GH+ group and GH- group. A total of 676 cycles were included in the present study with 338 cycles in each group. Conventional ovarian stimulation protocols were applied for ART treatment. Patients were further divided into POSEIDON group 3 (PG3, age <35 years) and POSEIDON group 4 (PG4, age ≥35 years), based on POSEIDON criteria. The demographic data, cycle characteristics, and clinical outcomes between the GH+ group and GH- group, as well as in the further stratified analysis of PG3 and PG4 were compared. GH adjuvant showed a beneficial effect on the ovarian response and live birth rate in poor ovarian reserve patients. Further stratification revealed that in PG4, there was a significantly increased number of good-quality embryos in the GH+ group compared to the GH- group (1.58 ± 1.71 vs. 1.25 ± 1.55, *P* = 0.032), accompanied by a reduced miscarriage rate and a greatly improved live birth rate (29.89 vs. 17.65%, *P* = 0.028). GH adjuvant failed to promote the live birth rate in PG3. In conclusion, GH pretreatment is advantageous by elevating ovarian response and correlated with an improved live birth rate and reduced miscarriage rate in POSEIDON poor ovarian reserve patients older than 35.

## Introduction

The incidence of low ovarian reserve among patients requiring assisted reproductive technology (ART) has been dramatically increasing. However, low ovarian reserve refers to depletion of the quantity and quality of oocytes in the ovary (1), and these patients may experience poor ovarian response, which is still a conundrum for clinicians (2). Many *in vitro* fertilization (IVF) centers supplement patients with various adjuvant therapies to enhance IVF success rates, such as growth hormone (3), coenzyme 10 (4), arginine (5), and dehydroepiandrosterone (6). The true beneficial effects of these therapies are actively debated (7). GH as an adjuvant therapy in IVF treatment has received most attention, such interest being resurrected by several interesting reports particularly since the mid-2000s (8, 9).

GH works through the somatotropic axis, which comprises GH, IGF-1, IGF-2 and their binding proteins and receptors. It has been proven to affect follicular recruitment directly or indirectly through insulin-like growth factor-1 (IGF-1) (10). The GH receptor has been shown to be expressed in theca cells, granular cells, and oocytes, both in animals and humans, providing physiological evidence for its efficacy in enhancing ovarian response and improving oocyte quality (11–13). Research on animals confirmed the indispensable role of GH in the various stages of follicular development, including follicle recruitment, development of preantral follicles, and gonadotropin sensitivity of antral follicles (11, 14, 15). GH binds to their receptors on granular cells resulting in enhanced proliferation and differentiation of the target cell (10). GH addition might elevate the density of FSH receptors in granulosa cells and increase the mitochondrial amount in human oocytes, which may further improve the ovarian response as well as the capacity to repair DNA mistakes (13, 16).

With these laboratory foundations, clinicians have increased confidence in GH adjuvant therapy for patients with POR, and many clinical studies have been performed. A prospective observational study of GH co-treatment with antagonist protocol reported advanced pregnancy and an increase in the number of high-quality embryos in the GH+ group (17). A recent multicentric randomized placebo-controlled trial, published by Norman et al. (18), was unable to demonstrate an increase in the live birth rate from the co-administration of GH in poor ovarian responders (PORs), but the study failed to reach its planned recruitment numbers (being only 130 instead of 390 cases). However, a large amount of literature did not reach a consensus on the effect of GH on PORs, with some papers reporting encouraging results (8, 9, 13, 19) and other articles posing negative consequences (20–22). There is no consensus on the dosage of GH treatment, the reported dosage is ranged from 1 IU every other day to 10 IU daily. 2 IU daily is the most economical and effective dose based on the combination of treatment experience in children with GH deficiency and IGF-1 levels among people of different ages, which is also confirmed in our previous study (9, 23, 24). In addition, the definition of POR is inconsistent and has more than 41 different visions in a recent meta-analysis enrolling 46 RCTs (25), which makes it difficult to determine the effect due to the heterogeneity of cases. Despite the recognized heterogeneity, there is a tendency to believe that GH addition may be beneficial for oocyte quality and thereby improving the live birth rate in the elderly subgroup.

Numerous definitions of POR in the past impede the consistency of research subjects in separate reports. In 2011, the European Society of Human Reproduction and Embryology working groups proposed the “Bologna Criteria” for POR (26). The effect of GH adjuvant in POR diagnosed by the “Bologna Criteria” still differs within the new research in that there are different durations and dosages of GH and mixed groups of patients. The shortcoming of this definition may be the very heterogeneity in patients with a disparate probability of successful conception (2), which was amended by the recently proposed POSEIDON (Patient-Oriented Strategies Encompassing IndividualizeD Oocyte Number) criteria for patients undergoing IVF (27). In short, patients were divided into two categories according to the POSEIDON criteria: patients with normal ovarian reserve (anti-Müllerian hormone [AMH] ≥ 1.2 ng/ml, antral follicular count [AFC] ≥5) and with unexpected poor or suboptimal ovarian response, patients with poor ovarian reserve (AMH <1.2 ng/ml, AFC <5) and with expected poor ovarian response. Both categories were further classified by age (POSEIDON group 1 [PG1] and PG3 <35 years, PG2 and PG4 ≥35 years) (27). The POSEIDON criteria stratifies low prognosis groups into more homogenous sub-groups and provides recommendations for clinical handling, which might be a better sorting scheme.

In the current retrospective report, we aimed to explore the efficiency of GH for patients in the specific cohort with poor ovarian reserve (AMH <1.2 ng/ml), including POSEIDON group 3 (PG3) and 4 (PG4). We examined whether GH could improve ART success rates by reducing miscarriage rates and thereby improving the live birth rate. Importantly, the study facilitates the exploration of the potential mechanism by which GH adjuvant may exert its benefits.

The study was approved by the Ethics Committee of the Sixth Affiliated Hospital of Sun Yat-Sen University (2016ZSLYEC-061).

## Materials and Methods

### Patients

Poor ovarian reserve patients (AMH <1.2 ng/ml), who met the PG3 or PG4 criteria, and underwent ART treatment in the Reproductive Medicine Center of The Sixth Affiliated Hospital of Sun Yat-Sen University from January 2014 to April 2016 were enrolled. In the GH+ group, 2 IU of GH in the form of Jintropin (Gensci, Changchun, China) was administered during the preceding menstrual cycle on days 2–3, which included daily injection over a 6-week period in the lead-up to ovum pick-up (OPU). Other enrolled patients without adjuvant treatment were included in the GH- group. The exclusion criteria were: abnormal chromosome, hydrosalpinx, endometriosis, hyperprolactinemia, thyroid diseases, uterine disorders that affected embryo implantation, severe oligoasthenozoospermia or azoospermia of the male partner. Among the cycles included, one-to-one case-control matching was performed to adjust essential confounding factors between the GH+ group and GH- group with SPSS 22.0, including age, AMH, body mass index (BMI) and AFC. A total of 338 cycles in the GH+ group and 338 cycles in the GH- group were enrolled in the data analysis. The demographic data, cycle characteristics and clinical outcomes of the GH+ group, were compared with their counterparts in the GH- group.

### Protocol for Controlled Ovarian Hyperstimulation (COH)

2 IU of GH daily in the form of Jintropin (Gensci, Changchun, China) was given subcutaneously on days 2–3 of the preceeding menstrual cycle until ovum pick-up (OPU) in the GH+ group. Conventional protocols, including both the antagonist and the long agonist protocol were applied in both groups (noting that the 2019 ESHER COS guideline indicated that these were equally effective for poor responders) (28). In the gonadotropin-releasing hormone (GnRH) antagonist protocol, gonadotropin (Gn) was administered from the second day of the cycle, and GnRH antagonist was administered subcutaneously daily when the leading follicle reached 14 mm until the day of hCG trigger. In the GnRH agonist protocol, 0.1 mg/day of leuprolide acetate was given subcutaneously from the midluteal phase of the previous cycle and Gn was administered 14 days later at the same time after achieving desensitization (FSH <5 IU/L, LH <5 IU/L, E2 <50 pg/ml) until the day of hCG administration. Ovidrel (Merck Serono, Germany) 0.25 mg was injected for the final trigger when dominant follicles reached 16 mm in diameter. Ultrasound-guided oocyte retrieval was performed ~36 h after the trigger.

Embryo culture was performed following standard protocols and scored by the international morphological grading system, Peter scoring system: grade 1, blastomeres are almost even with no particle cytoplasm, fragmentation rate is <5%; grade 2, blastomeres are slightly uneven with cytoplasm contained some particles, fragmentation rate is between 5 and 20%; grade 3, blastomeres are obviously uneven with obvious particles in cytoplasm, fragmentation rate is between 21 and 50% and grade 4 refers to embryos that blastomeres are severely uneven with severe particles in cytoplasm and the fragmentation rate is more than 50% (29). Cleavage embryo on day 3 with grades 1–3 and at least 5 blastomeres are considered as transferrable embryos, and cleavage embryo grades 1 or 2 with 6–10 blastomeres were considered as good quality embryos. Blastocysts were evaluated with the Gardner scoring system: grading stage 1–6 by the expansion and hatching of the blastocyst; rating A-C for the inter cell mass (ICM) and trophectoderm (TE) (30). Blastocysts on day 5/6 with stage 2–6 are considered to be embryos suitable for transfer, and scoring 3BB or higher are considered as good quality embryos. Fresh embryos were transferred either on day 3 at cleavage stage or on day 5 at blastocyst stage, no more than 2 embryos, were transferred. The luteal phase was supported by Utrogestan (Besins, France) 200 mg vaginally twice a day starting on the day of OPU.

### Data Collection and Outcome Measures

Data including age, duration of infertility, AFC, basal FSH, AMH, total Gn dosage, Gn duration, endometrium thickness, embryo development, and clinical outcomes were compared. Serum β-HCG levels >50 U/L at 12 days after blastocyst transplantation or at 14 days after cleavage stage embryo transplantation were confirmed as chemical pregnancies. Clinical pregnancy was identified by a gestational sac 3 weeks after a positive hCG test. The miscarriage rate was computed as the number of cycles that resulted in miscarriage by the number of clinical pregnancy cycles. The implantation rate was calculated as the number of gestational sacs divided by the number of embryos transferred. The live birth was defined as each live delivery of at least one fetus after 28 weeks of gestation.

### Statistical Analysis

A 1:1 case-control matching undertaken as a computer-generated exercise, was carried out to match the essential confounding parameters [age, AMH, body mass index (BMI), AFC] between the GH+ and GH-groups. The results are expressed as the mean ± standard deviation (SD) for numeric variables and the percentages for categorical variables, analyzed by SPSS version 22.0 (SPSS Inc., Chicago, IL, USA). The normality of the continuous variables was tested using the Kolmogorov–Smirnov test, and means were subsequently analyzed using either the two-tailed *t*-test (normal data distribution) or the Mann– Whitney *U*-test (skewed data) to compare two means where appropriate. Proportions were tested using the Chi-square test where appropriate. In all cases, statistical significance was established at *P* < 0.05.

## Result

A total of 676 cycles were enrolled in this study. Demographic data and cycle characteristics of all patients are summarized in Table 1. There was no significant difference in the basal demographic conditions between the GH+ group and the GH-group, in terms of age (36.96 ± 4.77 vs. 36.84 ± 4.74, *P* > 0.05), basal FSH (9.42 ± 5.66 vs. 8.78 ± 3.87, *P* > 0.05), AMH (0.69 ± 0.31 vs. 0.68 ±0.32, *P* > 0.05), and AFC (5.54 ± 2.55 vs. 5.60 ± 2.47, *P* > 0.05). Both groups underwent a similar composition of conventional protocols (*P* > 0.05). Patients in the GH+ group had previously suffered more failed attempts (2.63 ± 1.81 vs. 2.28 ± 1.99, *P* = 0.016) but were inclined to have a lower dosage and shorter duration of Gn stimulation, in keeping with the consistently recognized beneficial effect of GH co-treatment from the earliest studies in the 1980's (31). Finally, the number of oocytes retrieved (3.64 ± 2.83 vs. 3.54 ± 2.80, *P* > 0.05) was equivalent, and further culture resulted in an equal number of 2PN oocytes (2.39 ± 2.27 vs. 2.32 ± 2.21, *P* > 0.05), number of transferrable embryos (1.90 ± 1.95 vs. 1.83 ± 1.86, *P* > 0.05) and number of good quality embryos (1.53 ± 1.70 vs. 1.42 ± 1.59, *P* > 0.05) between the two groups. There were more cycles in the GH+ group than in the GH- group performed frozen only due to suboptimal endometrial features or personal reasons (144 vs. 76, *P* < 0.001), thus, fewer cycles in the GH+ group than in the GH- group had fresh embryos transferred (87 vs. 153, *P* < 0.001). However, the cancelation of fresh embryo transfers due to abnormal fertilization, unfertilized or no transferrable embryos in both groups are similar (107 vs. 109, *P* > 0.05).

**Table 1:** Demographic data and cycle characteristics of matched cycles with GH adjuvant (*n* = 676).

	**GH+ group** **(*n* = 338)**	**GH- group** **(*n* = 338)**	***P*-value**
Age (year)	36.96 ± 4.77	36.84 ± 4.74	0.725
Infertility years	4.96 ± 3.80	4.79 ± 3.48	0.832
BMI	22.66 ± 2.85	22.58 ± 2.72	0.770
Basal FSH (IU/L)	9.42 ± 5.66	8.78 ± 3.87	0.356
AMH (ng/ml)	0.69 ± 0.31	0.68 ± 0.32	0.746
AFC (*n*)	5.54 ± 2.55	5.60 ± 2.47	0.725
Failed attempts	2.63 ± 1.81	2.28 ± 1.99	<0.001
Cycles conducted with Long Protocol	179	188	0.487
Cycles conducted with Antangonsis Protocol	159	150	0.487
Gn dosage (IU)	2358.28 ± 625.87	2474.19 ± 695.07	0.039
Gn duration (day)	9.81 ± 2.13	10.73 ± 1.99	<0.001
Number of oocytes	3.64 ± 2.83	3.54 ± 2.80	0.726
Number of 2PN (n)	2.39 ± 2.27	2.32 ± 2.21	0.796
Number of transferable embryos (n)	1.90 ± 1.95	1.83 ± 1.86	0.789
Number of good quality embryos (n)	1.53 ± 1.70	1.42 ± 1.59	0.493
No. of canceled cycle that frozen all because of endometrium or patients' require	144	76	<0.001
No. of canceled cycle resulted in unfertilized or abnormal fertilized	107	109	0.869
Number of cycles with fresh embryo transfered	87	153	<0.001

*All values presented as mean ± SD*.

*P < 0.05 is considered statistically significant*.

*FSH, follicle-stimulating hormone; AMH, anti-Müllerian hormone; AFC, antral follicle count; Gn gonadotropin; 2PN, 2 pronuclei*.

In cycles that had fresh embryo transfer (data are shown in Table 2), there was no difference in demographic data, age, endometrial thickness, number of embryos transferred, proportion of embryonic development stage (cleavage/blastocyst), good quality embryo rate (81.38 vs. 78.89%, *P* > 0.05) or implantation rate (23.45 vs. 19.26%, *P* > 0.05). Surprisingly, the live birth rate per transfer cycle in the GH+ group was markedly higher than that of the GH- group (29.89 vs. 17.65%, *P* = 0.028), which might owe to significant lower miscarriage rate within the GH+ group (13.33 vs. 41.30%, *P* = 0.009). In order to further clarify whether the effect of GH on clinical results was related to patient age, we divided all the enrolled patients into POSEIDON group 3 (PG3, age <35 years old) or group 4 (PG4, age≥35 years old), and the results are presented in Table 3. All demographic data and clinical characteristics were comparable in the separate age groups. In PG3, younger patients were of equal age (31.35 ± 2.21 vs. 31.55 ± 2.27, *P* > 0.05), comparable basal FSH (9.73 ± 6.67 vs. 8.85 ± 3.80, *P* > 0.05), AMH (0.71 ± 0.31 vs. 0.69 ± 0.32, *P* > 0.05), and AFC (5.84 ± 2.97 vs. 6.15 ± 2.77, *P* > 0.05) between the GH+ group and GH- group. Patients in the GH+ group had a higher BMI than patients in the GH- group (22.01 ± 2.67 vs. 21.39 ± 2.57, *P* = 0.033). Further analysis of the difference according to the patients' BMI: lean (BMI <18.5 kg/m2); normal (18.5 kg/m2 ≤ BMI <25 kg/m2); and overweight (BMI≥25 kg/m2), no significant difference in the subgroups distribution of patients was found between the two groups (*P* > 0.05). However, GH adjuvant showed a beneficial effect on the ovarian response, as the total Gn dosage was significantly lower (2351.39 ± 642.95 vs. 2577.80 ± 704.70, *P* = 0.013) with shorter Gn stimulation duration (9.69 ± 2.33 vs. 10.78 ± 1.95, *P* < 0.001) in GH+ group compared to GH- group while the composition of two protocols are similar between these groups (*P* > 0.05). Furthermore, 2019 ESHRE COS guideline has indicated that antagonist and agonist protocol are equally effective for poor responder, which could help us to better explain our results (28). A similar number of oocytes was retrieved (3.87 ± 2.76 vs. 4.40 ± 3.05, *P* > 0.05) and good quality embryos developed (1.42 ± 1.69 vs. 1.75 ± 1.61, *P* > 0.05), although the number of 2PN (2.39 ± 2.34 vs. 2.95 ± 2.39, *P* = 0.025) and transferrable embryos (1.79 ± 1.88 vs. 2.20 ± 1.87, *P* = 0.034) were fewer.

**Table 2:** Cycle characteristic and clinical outcomes of fresh embryo transferred cycles (*n* = 240).

	**GH+ group** **(*n* = 87)**	**GH- group** **(*n* = 153)**	***P*-value**
Age (year)	36.25 ± 4.20	35.89 ± 4.87	0.538
Endometrial thickness (mm)	11.02 ± 2.07	11.22 ± 2.43	0.699
Number of embryo transferred	1.67 ± 0.47	1.77 ± 0.44	0.699
Number of transferred embryos on Day 3	78	135	0.738
Number of transferred embryos on Day 5/6	9	18	0.738
Good quality embryo rate in transferred embryos (%)	81.38% (118/145)	78.89% (213/270)	0.547
Biochemical pregnancy rate (%)	36.78% (32/87)	36.60% (56/153)	0.978
Clinical pregnancy rate (%)	35.63% (30/87)	32.68% (46/153)	0.479
Miscarriage rate (%)	13.33% (4/30)	41.30% (19/46)	0.009
Implantation rate (%)	23.45% (34/145)	19.26% (52/270)	0.607
Twin pregnancy rate per transfer cycle (%)	4.60% (4/87)	3.92% (6/153)	0.801
Ectopic pregnancy rate per transfer cycle (%)	1.15% (1/87)	2.61% (4/153)	0.656
Live delivery rate per transfer cycle (%)	29.89% (26/87)	17.65% (27/153)	0.028

All values presented as mean ± SD.

*P < 0.05 is considered statistically significant*.

**Table 3:** Demographic data and cycle characteristics of cycles with GH adjuvant in PG3 and PG4 (*n* = 676).

	**PG3 (age < 35 years)**			**PG4 (age** ≥ **35 years)**		
	**GH+ group (*n* = 108)**	**GH- group (*n* = 116)**	***P*-value**	**GH+ group (*n* = 230)**	**GH- group (*n* = 222)**	***P*-value**
Age (year)	31.35 ± 2.21	31.55 ± 2.27	0.340	39.60 ± 3.07	39.60 ± 3.04	0.997
Infertility years	3.91 ± 2.58	4.23 ± 2.21	0.189	5.46 ± 4.16	5.08 ± 3.96	0.351
BMI	22.01 ± 2.67	21.39 ± 2.57	0.033	22.96 ± 2.89	23.19 ± 2.58	0.221
lean	9	7	0.635	9	2	0.114
normal	82	94	–	175	173	–
overweight	17	15	–	46	47	–
Basal FSH (IU/L)	9.73 ± 6.67	8.85 ± 3.80	0.756	9.25 ± 5.07	8.75 ± 3.91	0.427
AMH (ng/ml)	0.71 ± 0.31	0.69 ± 0.32	0.737	0.68 ± 0.30	0.67 ± 0.32	0.879
AFC (n)	5.84 ± 2.97	6.15 ± 2.77	0.264	5.40 ± 2.32	5.32 ± 2.25	0.653
Failed attempts	2.58 ± 1.79	1.83 ± 1.51	<0.001	2.65 ± 1.83	2.51 ± 2.17	0.041
Cycles conducted with Long Protocol	62	81	0.053	117	107	0.570
Cycles conducted with Antangonsis Protocol	46	35	0.053	113	115	0.570
Gn dosage (IU)	2351.39 ± 642.95	2577.80 ± 704.70	0.005	2361.52 ± 619.09	2422.05 ± 685.36	0.526
Gn duration (day)	9.69 ± 2.33	10.78 ± 1.95	<0.001	9.87 ± 2.03	10.71 ± 2.02	<0.001
Number of occytes	3.87 ± 2.76	4.40 ± 3.05	0.139	3.53 ± 2.86	3.09 ± 2.56	0.130
Number of 2PN (n)	2.39 ± 2.34	2.95 ± 2.39	0.025	2.39 ± 2.24	2.00 ± 2.03	0.036
Number of transferable embryos (n)	1.79 ± 1.88	2.20 ± 1.87	0.034	1.95 ± 1.98	1.64 ± 1.82	0.055
Number of good quality embryos (n)	1.42 ± 1.69	1.75 ± 1.61	0.053	1.58 ± 1.71	1.25 ± 1.55	0.018

*All values presented as mean ± SD*.

*P < 0.05 is considered statistically significant*.

*FSH, follicle-stimulating hormone; AMH, anti-Müllerian hormone; AFC, antral follicle count; Gn gonadotropin; 2PN, 2 pronuclei*.

In the PG4 group, elderly patients were also of comparable age (39.60 ± 3.07 vs. 39.60 ± 3.04, *P* > 0.05) and equivalent ovarian reserve status between the GH+ and GH- groups, as the basal FSH (9.25 ± 5.07 vs. 8.75 ± 3.91, *P* > 0.05), AMH (0.68 ± 0.30 vs. 0.67 ± 0.32, *P* > 0.05), and AFC (5.40 ± 2.32 vs. 5.32 ± 2.25, *P* > 0.05) were well-matched. Similarly, the GH adjuvant improved the ovarian response by decreasing the duration of Gn stimulation (9.87 ± 2.03 vs. 10.71 ± 2.02, *P* < 0.001) with similar protocols. The number of oocytes retrieved (3.53 ± 2.86 vs. 3.09 ± 2.56, *P* > 0.05) was equal between the two groups. Further culture with a parallel number of oocytes, showed a significant increase in the number of good-quality embryos (1.58 ± 1.71 vs. 1.25 ± 1.55, *P* = 0.032), indicating that the oocyte utilization rate was greatly increased with improved embryo quality.

Clinical outcomes are further analyzed as well in Table 4. No matter whether in the GH+ group or the GH- group, there were more patients in PG4 compared with PG3, which is in line with the realistic incidence of ovarian reserve decline being higher in elderly patients. In both PG3 and PG4, there was no difference in age (*P* > 0.05 in PG3 and *P* > 0.05 in PG4), endometrial thickness (*P* > 0.05 in PG3 and *P* > 0.05 in PG4), number of embryo transferred (*P* > 0.05 in PG3 and *P* > 0.05 in PG4), embryonic development, and the proportion of good quality embryo transferred (*P* > 0.05 in PG3 and *P* > 0.05 in PG4) between the GH+ and GH- groups. In PG3 and PG4, clinical pregnancy rate, implantation rate, twin pregnancy rate, and ectopic pregnancy rate were equal between the GH+ group and the GH- group. Although GH did not reveal any beneficial in terms of biochemical pregnancy rate, GH supplement in PG4 achieved a borderline improved clinical pregnancy rate (36.7 vs. 23.0%, *P* = 0.071) and a significant increase in live birth rate (27.3 vs. 9.2%, *P* = 0.003), accompanied with decrease in miscarriage rate significantly (18.2 vs. 60.0%, *P* = 0.005).

**Table 4:** Cycle characteristic and clinical outcomes of fresh embryo transferred cycles in PG3 and PG4 (*n* = 240).

	**PG3(age < 35 years)**			**PG4 (age** ≥ **35 years)**		
	**GH+ group (*n* = 27)**	**GH- group (*n* = 66)**	***P*-value**	**GH+ group (*n* = 60)**	**GH- group (*n* = 87)**	***P*-value**
Age (year)	31.30 ± 1.75	31.33 ± 2.38	0.569	38.48 ± 2.85	39.35 ± 3.11	0.095
Endometrial thickness (mm)	11.33 ± 1.80	11.96 ± 2.11	0.155	10.82 ± 2.23	10.70 ± 2.48	0.443
Number of embryos transferred	1.74 ± 0.45	1.83 ± 0.38	0.308	1.63 ± 0.49	1.71 ± 0.48	0.345
Number of transferred embryos on Day 3	26	61	0.668	52	74	0.784
Number of transferred embryos on Day 5/6	1	5	0.668	8	13	0.784
Good quality embryo rate in transferred embryos (%)	78.7% (37/47)	80.2% (97/121)	0.833	82.7% (81/98)	73.8% (110/149)	0.105
Biochemical pregnancy rate (%)	29.6% (8/27)	51.5% (34/66)	0.054	40.0% (24/60)	25.3% (22/87)	0.059
Clinical pregnancy rate (%)	29.6% (8/27)	39.4% (26/66)	0.375	36.7% (22/60)	23.0% (20/87)	0.071
Miscarriage rate (%)	0% (0/8)	26.9% (7/26)	0.100	18.2% (4/22)	60.0% (12/20)	0.005
Implantation rate (%)	76.9% (10/47)	24.0% (29/121)	0.711	24.5% (24/98)	15.4% (23/149)	0.076
Twin pregnancy rate per transfer cycle (%)	7.4% (2/27)	4.5% (3/66)	0.626	3.0% (2/66)	3.4% (3/87)	1.000
Ectopic pregnancy rate per transfer cycle (%)	0.0% (0/27)	6.1% (4/66)	0.319	1.5% (1/66)	0.0% (0/87)	0.431
Live delivery rate per transfer cycle (%)	29.6% (8/27)	28.8% (19/66)	0.935	27.3% (18/66)	9.2% (8/87)	0.003

*All values presented as mean ± SD*.

*P < 0.05 is considered statistically significant*.

*No, Number*.

## Discussion

In this retrospective study based on POSEIDON criteria, poor ovarian reserve patients were enrolled and further classified as PG3 and PG4. The results revealed that GH adjuvant during COH benefits the ovarian response, and promotes the clinical outcome of patients over 35 years old who underwent IVF/ICSI treatment. These results were similar to our previous self-controlled research, but demonstrated a more specific subgroup of patients (9). In a study by Cochrane, GH administration helped improve the live birth rate of PORs, but the research did not define the subgroups of POR patients who actually benefited from the GH adjuvant (32). Before the POSEIDON criteria were posed, the Bologna criteria were once popular. However, the Bologna criteria have a fuzzy definition of the threshold of ovarian reserve markers (i.e., AFC <5–7 follicles or AMH <0.5–1.1 ng/mL) and of “other cause in POR” (26). In addition, it brings the risk of categorizing patients with significant differences in biological characteristics (33). Interestingly, our studies were conducted only during the time of diagnostic criteria reform. In our previous study, we enrolled patients with POR diagnosed by the Bologna criteria. Despite the heterogeneity of patients, we conducted it with a self-control design to minimize the heterogeneity, resulting in a significant positive conclusion (9). Along with the progress in POR criteria, we carried out this study with the aim specifying the subgroup in which GH could be the most beneficial. We enrolled patients who complied with the definition of PG3 and PG4 (AMH <1.2 ng/ml), avoiding interobserver differences in AFC.

Among all enrolled patients with poor ovarian reserve, lower dosages and shorter durations of Gn stimulation were detected in the GH+ group of all POR patients, which implies an enhancement in ovarian response, illustrating the important role of GH in the proliferation and differentiation of granulosa cells, as demonstrated in animal research (14). In further analysis, GH addition promoted the number of good quality embryos, clinical pregnancy rate, and live birth rate in women with poor ovarian reserves who were older than 35 years. Importantly, encouraging results in the GH+ group also echoed our previous work (9), the reported data by Yovich et al. (8, 34) and data revealed by Tesarik et al. (35) in a RCT. The homogeneity of patients was better in our research given that Yovich et al. enrolled patients diagnosed with broad Bologna criteria and Tesarik et al. recruited patients with a smaller range of ages. Although a recent multicentric randomized placebo-controlled trial, published by Norman et al. (18), provided no evidence for an increase in the live birth rate from the large dosage GH co-treatment and not recommended for widespread use in PORs, the patients enrolled were in a broader criteria in that the study was designed much earlier than the newly classification proposed. Furthermore, that study was limited by recruitment failure, a feature acknowledged by the authors. However, they also believed that the currently recognized definition such as POSEIDON may have unmasked a subgroup of PORs that can really benefit from GH. Though the live birth is multifactorial, the quantity and quality of oocytes equally contribute to pregnancy outcomes in women with POR and age is the only predictor of quality available (36). As ovarian reserve is irreversible, GH addition may increase oocyte quality as well as ovarian response, especially in aged patients with poor ovarian reserve, thus increasing the live birth rate of these patients (37).

Patients younger than 35 years old in the GH+ group had higher BMI compared to patients in the GH- group, however, we reanalyzed the difference according to the patients' BMI and found no significant difference in the subgroup distribution of patients between the three groups (*P* > 0.05). They were treated with significantly lower total Gn dosages and shorter Gn stimulation durations. This economic effect of reducing the total Gn dosage and duration was shown among all enrolled patients, which implies that GH adjuvant promoted the ovarian response, which is a different conclusion compared with our previous study (9). Besides, Ahmed et al. has reported that there was no significant difference among poor responders with different BMI in gonadotropin dose, duration of stimulation, number of oocytes retrieved, number of embryos, transferred embryos in a prospective cohort study (38), which may provide indirect evidence that GH administration improve ovarian response in patients younger than 35 years old. Although a comparable number of oocytes was collected, fewer 2PN and transferrable embryos were formed. However, this difference failed to reach statistical significance in number of good quality embryos, causing us to reconsider the value of GH in young patients. Another study focusing on young patients is urgently needed.

Development in laboratory research in older women provides a theoretical basis for our study. Mitochondria are considered to be a keystone of oocyte development potential, but both the quality and the quantity of mitochondria and mtDNA number in oocytes are significantly decreased with female aging, and the addition of GH could partially amend these features (13). It has recently been reported that GH co-treatment in older patients with reduced ovarian reserve can modulate the density of GH receptors in granulosa cells and further improve clinical outcome (39).

This study has its limitation as a retrospective analysis, but it still provides important clues aiming to improve therapeutic intervention strategies for POR patients. It is the first paper based on the POSEIDON criteria to distinguish specific subgroups of POR that GH works effectively, which may clarify the detailed adjuvant methods and specific subgroups patients of GH treatment and avoiding extra economic burden for patients who are invalid. Molecular marker detection is still required to further support our results in a well-designed, multicenter, prospective RCT.

Taken together, 2 IU of GH adjuvant ~6 weeks preceding OPU is sufficient to reveal the beneficial effects of GH on promoting the live birth rate for PG4 patients diagnosed by POSEIDON criteria.

## Data Availability Statement

The datasets generated for this study are available on request to the corresponding author.

## Ethics Statement

The studies involving human participants were reviewed and approved by 2016ZSLYEC-061. Written informed consent for participation was not required for this study in accordance with the national legislation and the institutional requirements.

## Author Contributions

XY conceived and designed the study. MC made substantial contributions to data analysis and interpretation. LG and YW completed the data collection. XY, XL, and CF reviewed the content and critically revised the manuscript. All authors contributed to writing the manuscript.

### Conflict of Interest

The authors declare that the research was conducted in the absence of any commercial or financial relationships that could be construed as a potential conflict of interest.
